# Household mold exposure interacts with inflammation-related genetic variants on childhood asthma: a case–control study

**DOI:** 10.1186/s12890-021-01484-9

**Published:** 2021-04-02

**Authors:** Yu Zhang, Li Hua, Quan-Hua Liu, Shu-Yuan Chu, Yue-Xin Gan, Min Wu, Yi-Xiao Bao, Qian Chen, Jun Zhang

**Affiliations:** 1 Ministry of Education-Shanghai Key Laboratory of Children’s Environmental Health, School of Public Health, Shanghai Jiao Tong University School of Medicine, Shanghai, China; 2 Ministry of Education-Shanghai Key Laboratory of Children’s Environmental Health, Xinhua Hospital, Shanghai Jiao Tong University School of Medicine, 1665 Kongjiang Road, Shanghai, 200092 China; 3 Laboratory of Respiratory Disease, Affiliated Hospital of Guilin Medical University, Guilin, Guangxi China; 4 Department of Pediatric Pulmonology, Xinhua Hospital, Shanghai Jiao Tong University School of Medicine, 1665 Kongjiang Road, Shanghai, 200092 China; 5 Department of Chinese Traditional Medicine, Xinhua Hospital, Shanghai Jiao Tong University School of Medicine, 1665 Kongjiang Road, Shanghai, 200092 China

**Keywords:** Mold, Additive interaction, Rs7216389, Single nucleotide polymorphisms, Childhood asthma

## Abstract

**Background:**

A number of studies have examined the association between mold exposure and childhood asthma. However, the conclusions were inconsistent, which might be partly attributable to the lack of consideration of gene function, especially the key genes affecting the pathogenesis of childhood asthma. Research on the interactions between genes and mold exposure on childhood asthma is still very limited. We therefore examined whether there is an interaction between inflammation-related genes and mold exposure on childhood asthma.

**Methods:**

A case–control study with 645 asthmatic children and 910 non-asthmatic children aged 3–12 years old was conducted. Eight single nucleotide polymorphisms (SNPs) in inflammation-related genes were genotyped using MassARRAY assay. Mold exposure was defined as self-reported visible mold on the walls. Associations between visible mold exposure, SNPs and childhood asthma were evaluated using logistic regression models. In addition, crossover analyses were used to estimate the gene-environment interactions on childhood asthma on an additive scale.

**Results:**

After excluding children without information on visible mold exposure or SNPs, 608 asthmatic and 839 non-asthmatic children were included in the analyses. Visible mold exposure was reported in 151 asthmatic (24.8%) and 119 non-asthmatic children (14.2%) (aOR 2.19, 95% CI 1.62–2.97). The rs7216389 SNP in gasdermin B gene (GSDMB) increased the risk of childhood asthma with each C to T substitution in a dose-dependent pattern (additive model, aOR 1.32, 95% CI 1.11–1.57). Children carrying the rs7216389 T allele and exposed to visible mold dramatically increased the risk of childhood asthma (aOR 3.21; 95% CI 1.77–5.99). The attributable proportion due to the interaction (AP: 0.47, 95% CI 0.03–0.90) and the relative excess risk due to the interaction (RERI: 1.49, 95% CI 0–2.99) were statistically significant.

**Conclusions:**

In the present study, there was a significant additive interaction between visible mold exposure and rs7216389 SNP on childhood asthma. Future studies need to consider the gene-environment interactions when exploring the risk factors of childhood asthma.

**Supplementary Information:**

The online version contains supplementary material available at 10.1186/s12890-021-01484-9.

## Introduction

Asthma is a common chronic airway inflammatory disorder, characterized by hyper responsiveness, obstruction and chronic inflammation of the airway [1]. It has emerged as a major global public health problem. In 2016, the Global Burden of Disease (GBD) study estimated that 339.4 million people worldwide were affected by asthma, which has increased by 3.6% since 2006 [2]. The asthma epidemic experienced by high-income countries over the past 30 years has now become an increasing problem in low- and middle-income countries with the economic development and urbanization. As one of the largest middle-income countries, China has undergone changes with an unprecedented speed of urbanization, leading to changes in environment and child lifestyle. Meanwhile, the prevalence of childhood asthma has been increasing rapidly [3, 4]. The Third Nationwide Survey of Childhood Asthma in Urban Areas of China (2010) found that the prevalence of asthma in children under 14 years had increased from 2.0% in 2000 to 3.0%, and Shanghai had the highest rate of 7.6% [2].

The genetic susceptibility of asthma has been demonstrated by numerous studies using candidate gene associations and genome-wide associations (GWAS) method [5, 6]. Single nucleotide polymorphisms (SNPs) in inflammation-related genes have been shown to influence the development of childhood asthma. However, the results are inconsistent and inconclusive, even for the most replicated genes [7], probably due to the influence of environmental factors, which play an important role in the physiological pathways between genes and childhood asthma [8].

The urbanization has led people, especially children, spending most of their time in the indoor environment [9]. Evidence shows that the indoor environment plays a key role in the development of childhood asthma [10–13]. Visible mold, a common indoor dampness phenomenon, has been found closely associated with childhood asthma. For example, a meta-analysis with 31,742 children from eight European birth cohorts found that visible mold exposure during the first 2 years of life was positively associated with childhood asthma [14]. Two large epidemiologic studies from China showed that visible mold on walls was significantly associated with physician-diagnosed childhood asthma in both boys and girls [15, 16].

Shanghai, located at the Yangtze River estuary in the East China Sea, has a typical subtropical monsoon climate. Visible mold in buildings is common and frequent [17]. The surface pathogen-associated molecular patterns (PAMPs) in mold could induce distinct inflammatory phenotypes in the lungs, and increase the risk of asthma development and exacerbation [18]. Meanwhile, inflammation-related genes are involved in pro- and anti-inflammatory effects, and could modify the pathogenesis of asthma caused by mold exposure. Thus, we sought to investigate whether there is any gene-environment interaction between the inflammation-related gene polymorphisms and visible mold exposure in childhood asthma in Shanghai.

## Material and methods

### Study population and design

From June 2015 to January 2016, we conducted a case–control study aiming to explore the risk factors of childhood asthma. The design, recruitment and the characteristics of the study population have been previously described elsewhere [19]. Briefly, children aged 3 to 12 years with asthma were recruited in the case group from the Xinhua Hospital, Shanghai, China. Children with a history of recurrent wheezing (> 2 times) were examined by a physician, including medical history, physical exams and tests. According to the Global Initiative for Asthma (GINA) guidelines, asthmatic children were defined by history of recurrent wheezing, feeling of tightness or pain in the chest, cough, and with positive bronchial provocation (forced expiratory volume in one second (FEV1) > 200 ml after inhaling bronchodilator). The control group consisted of non-asthmatic children of the same age, from the pediatric outpatient clinic and pediatric surgery clinic of the same hospital. 645 asthmatic and 910 non-asthmatic children were recruited in our study. The study protocol was approved by the Institutional Review Board of the Xinhua Hospital (approval number: XHEC-C-2014-065), and conducted according to the principles in the Declaration of Helsinki. The informed consents were signed by all parents.

### Questionnaires

A face-to-face interview was conducted with the parents of the participants. The questionnaire included information on parental demographic factors, delivery mode of the child, feeding habits, and indoor environment including visible mold and environmental tobacco smoke (ETS) exposure. Mold exposure was assessed by the following question “Have you ever seen visible mold on your walls, ceiling, or floor in your home?”.

### Genotyping

Oral mucosal swabs were collected at recruitment and maintained at -80 ºC immediately after transportation to the biobank. Genomic DNA was isolated from oral mucosal swabs using the DNA extraction kit (Shanghai Lifefeng Biotechnology Co., China) according to the manufacturer’s manual. For the SNP selection, we reviewed the literature in PubMed. First, the candidate SNP was frequently linked to childhood asthma and associated with the inflammatory response. Second, at least one study has reported that the SNP interacted with environmental exposures such as mold, ETS, or pet exposure on the development of asthma. Eventually, eight SNPs were selected (information on the SNPs were shown in Additional file 1: Table S1). All SNPs were genotyped using the MassARRAY assay based on matrix-assisted laser desorption/ionization time-of-flight (MALDI-TOF) mass spectrometry platform (Agena Bioscience, USA), following the manufacturer’s instruction [20]. In each 384-well reaction plate, one positive and one negative control were added as quality control. Our primer and probe sequences were listed in Additional file 1: Table S2. All assays were performed by laboratory technicians blinded to case status.

To validate the accuracy and reliability of genotyping results by using MassARRAY assay, the genotyping results were verified by two steps. First, 40 (2.5%) samples were randomly selected to sequence using 3730xl DNA Sequencer (Applied Biosystems, Foster City, USA). No inconsistencies were observed. Second, 81 (5%) randomly selected samples were re-genotyped by using MassARRAY assay, and the concordance reached 99.7%.

### Covariates

Information on covariates was obtained from the questionnaire. A directed acyclic graph (DAG) was drawn to identify the potential confounding factors (see Additional file 1: Fig. S1). They were age, gender, parental education level, and family history of allergy. In addition, ETS exposure was also included as a confounder because it was a common cause of childhood asthma and frequently adjusted in similar studies [16, 21]. ETS exposure was defined as one or both parents smoked (yes) and none of parents smoked (no). Parental education level was categorized as: middle school or below, high school or technical school, college degree, and graduate degree or above. Missing data of the covariates were included as a separate category in the analyses.

### Statistical analyses

The differences in demographic characteristics, environmental factors and genotype frequencies of SNPs between the cases and controls were calculated using chi-square test for categorical variables, Student’s t-test or Wilcoxon test for continuous variables. The Hardy–Weinberg equilibrium (HWE) of allele frequencies in the control group was assessed by the goodness-of-fit chi-square. Odds ratios (ORs) and 95% confidence intervals (CIs) were calculated using logistic regression models to evaluate the associations between SNPs and childhood asthma under assumption of different genetic models, including additive (AA vs. AB vs. BB), dominant (AA + AB vs. BB) and recessive (AA vs. AB + BB) models (A represents mutant allele and B represents wild type allele). In addition, crossover analyses were used to assess the additive interactions between visible mold exposure and SNPs under dominant and recessive models. We assumed that G and E stand for risk genotype and environmental factor, with their absence and presence defined as 0 and 1, respectively. Additional file 1: Table S3 showed the basic research units in a 2 × 4 crossover analysis of the interaction between G and E, indicating the four possible combinations formed by the two binary variables. OR of being exposed to both G and E was labeled as OR_11_ representing joint effect. OR of being exposed only to G or E were labeled as OR_10_ and OR_01_, respectively, representing independent effects of G and E. All the ORs were estimated with G = 0 and E = 0 as the reference group (OR_00_ = 1) [22, 23]. In the crossover analyses, all ORs were adjusted for the aforementioned potential confounders. Relative excess risks due to interaction (RERI), the synergy index (SI), and attributable proportion of interaction (AP) proposed by Rothman were used to evaluate additive interaction effects [24]. AP > 0, RERI > 0, or S > 1 indicated additive interaction. In general, the OR is a good estimate of the risk ratio (RR) if the disease is rare (prevalence less than 10%) [25], then the calculation formulas for a case–control study were defined as follows: RERI = OR_11_—OR_01_—OR_10_ + 1; AP = RERI/OR_11_ and SI = (OR_11_—1)/((OR_01_—1) + (OR_10_—1)). CIs of the three additive interaction measures were calculated basing on the delta method described by Hosmer and Lemeshow [26].

The major and minor alleles were referred as wild and mutant alleles, respectively. For rs7216389 SNP, minor allele was referred to as wild allele due to lower risk effect of childhood asthma [27]. False discovery rate (FDR) adjusted *p* values were calculated to correct for multiple comparisons. All the analyses were performed in the statistical software package R (version 3.6.0). FDR adjusted *p* value < 0.05 was considered as statistical significance.

## Results

### Study population and genotyping

A total of 1555 children were genotyped for the eight SNPs in inflammation-related genes. Children without information on visible mold exposure (n = 91) or SNPs (n = 17) were excluded, leaving 608 asthmatic and 839 non-asthmatic children for analysis.

Characteristics of the cases and controls were presented in Table 1. The cases were younger and had more boys than the controls. Parental education level was also higher among the cases.

**Table 1:** Characteristics of the study population

	Asthma cases (N = 608)	Non-asthma controls (N = 839)
Age (y), mean (SD)	6.1 (2.0)	6.6 (2.1)
BMI, median (IQR)	15.8 (2.9)	15.7 (3.4)
Birthweight, mean (SD)	3309 (489)	3276 (507)
Gestational weeks at birth, median (IQR)	39 (2)	39 (1)
Maternal age at birth (y), mean (SD)	28.6 (3.5)	27.9 (3.9)
*Maternal educational level, (n, %)*		
≤ 9 years	40 (6.6)	138 (16.4)
10–12 years	79 (13)	151 (18)
13–16 years	395 (65)	443 (52.8)
> 16 years	49 (8.1)	45 (5.4)
*Paternal educational level, (n, %)*		
≤ 9 years	38 (6.2)	125 (14.9)
10–12 years	74 (12.2)	153 (18.2)
13–16 years	383 (63)	437 (52.1)
> 16 years	68 (11.2)	66 (7.9)
*Gender, (n, %)*		
Boy	368 (60.5)	454 (54.1)
Girl	240 (39.5)	385 (45.9)
*Family history of allergy, (n, %)*		
No	275 (45.2)	627 (74.7)
Yes	314 (51.6)	194 (23.1)
*Delivery mode, (n, %)*		
Vaginal	211 (34.7)	328 (39.1)
Caesarean section	394 (64.8)	507 (60.4)
*Exclusive breastfeeding, (n, %)*		
< 6 months	301 (49.5)	426 (50.8)
≥ 6 months	305 (50.2)	413 (49.2)
*Carpet in the dwelling, (n, %)*		
No	562 (92.4)	786 (93.7)
Yes	37 (6.1)	41 (4.9)
*Pet in the dwelling, (n, %)*		
No	539 (88.7)	740 (88.2)
Yes	58 (9.5)	94 (11.2)
*ETS at birth, (n, %)*		
No	430 (70.7)	547 (65.2)
Yes	172 (28.3)	289 (34.4)
*ETS at present, (n, %)*		
No	421 (69.2)	521 (62.1)
Yes	178 (29.3)	312 (37.2)
*Visible mold in the dwelling, (n, %)*		
No	457 (75.2)	720 (85.8)
Yes	151 (24.8)	119 (14.2)

*SD* standard deviation, *IQR* inter-quartile range, *ETS* environmental tobacco smoke

The reference sequence (rs) numbers, minor allele frequencies, and HWE tests of SNPs included in the present study were shown in Additional file 1: Table S4. All the SNPs were common polymorphisms with minor allele frequencies (MAF) of 10–48% and were in Hardy–Weinberg equilibrium (*p* > 0.12).

### Environmental exposure

In total, 24.8% (151/608) of the cases and 14.2% (119/839) of the controls were exposed to visible mold, respectively. After adjusting for age, gender, family history of allergy, parental education level, and ETS exposure before and after birth, visible mold exposure was also significantly associated with a higher risk of childhood asthma (aOR 2.19, 95% CI 1.62–2.97).

### Associations between inflammation-related genetic polymorphisms and childhood asthma

We evaluated the associations between the SNPs and childhood asthma in different genetic models. Under the recessive model, comparing to subjects carrying C allele of the rs7216389, subjects with the homozygous TT genotype had a significantly increased risk of childhood asthma (TT vs. CC + TC, aOR 1.34, 95% CI 1.08–1.66, P-FDR: 0.06), which remained borderline significant after adjusting for multiple comparisons. The risk effect was stronger under the dominant model (TT + TC vs. CC, aOR 1.72, 95% CI 1.09–2.77, P-FDR: 0.18). Moreover, a dose-dependent association was found under the additive model, with 1.32-fold increased risk per more T allele (aOR 1.32, 95% CI 1.11–1.57, P-FDR: 0.02) (Table 2). There was no significant correlation between other SNPs and childhood asthma (See Additional file 1: Table S5).

**Table 2:** Association between rs7216389 polymorphism and childhood asthma under different genetic models

Genetic model	Genotype	aOR (95% CI)	FDR adjusted *p* value
Dominant	CC	ref	0.18
	TT + TC	1.72 (1.09, 2.77)	
Recessive	CC + CT	ref	0.06
	TT	1.34 (1.08, 1.66)	
Additive ^a^	T allele	1.32 (1.11, 1.57)	0.02

*aOR* adjusted odds ratio, *Ref* reference, *FDR* False discovery rate

^a^The genotypes were categorized into a three-level variable for the number of major alleles under the additive model (0, 1, 2)

Models were adjusted for age and gender

### Additive effects of SNPs and visible mold exposure on childhood asthma

**Fig. 1:**
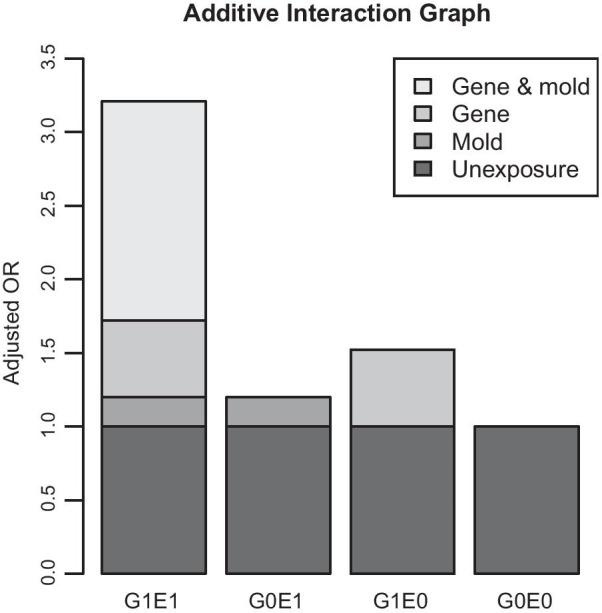
Additive effect of visible mold exposure and rs7216389 SNP on childhood asthma under the dominant model (TT + TC vs. CC). Definition of abbreviations: SNP = single nucleotide polymorphisms; G = risk genotype, 0 and 1 represent absence and presence of the factor, respectively; E = environmental factor, 0 and 1 represent absence and presence of the factor, respectively; OR = odds ratio. “G0E0”, “G1E0”, “G0E1” and “G1E1” represented four combinations of G and E exposure. Gene & mold represented the relative excess risk due to interaction between rs7216389 polymorphism and visible mold exposure on childhood asthma

Compared to non-exposed children who carried rs7216389 CC genotype, the relative risk of childhood asthma in non-exposed subjects who carried T allele was 1.52 (95% CI 0.88–2.70). In contrast, the same genotypes in children exposed to visible mold were associated with a significantly increased risk of childhood asthma (aOR_11_: 3.21, 95% CI 1.77–5.99, P-FDR: < 0.001), which was greater than the sum of their independent effects (Fig. 1). However, visible mold exposure was not significantly associated with asthma in children carrying the CC genotype (aOR_01_: 1.20, 95% CI 0.38–3.65). The relative excess risk contributed by the additive interaction between the rs7216389 risk genotypes and visible mold exposure was 1.49 (95% CI 0–2.99). Furthermore, the proportion of childhood asthma attributable to the interaction was as high as 47% (AP: 0.47, 95% CI 0.03–0.90) (Table 3).

**Table 3:** Combined effects of selected SNPs and visible mold exposure on childhood asthma under the dominant model

SNP/visible mold exposure	No. of asthma cases	Group size	aOR (95% CI)	AP	S	RERI
*rs1042713/mold*				− 0.09 (− 0.70, 0.53)	0.86 (0.32, 2.35)	− 0.19 (− 1.51, 1.13)
AA/no	148	398	Ref			
GG + GA/no	309	779	1.13 (0.87, 1.47)			
AA/yes	42	73	2.23 (1.30, 3.86)*			
GG + GA/yes	109	197	2.17 (1.50, 3.15)*			
*rs1042714/mold*				0.10 (− 0.51, 0.70)	1.23 (0.32, 4.70)	0.20 (− 1.15, 1.55)
CC/no	376	958	Ref			
GG + GC/no	81	219	0.91 (0.65, 1.25)			
CC/yes	120	217	1.96 (1.43, 2.70)*			
GG + GC/yes	31	53	2.07 (1.15, 3.79)*			
*rs7216389/mold*				0.47 (0.03, 0.90)	3.08 (0.36, 26.06)	1.49 (0, 2.99)
CC/no	21	69	Ref			
TT + TC/no	436	1108	1.52 (0.88, 2.70)			
CC/yes	7	20	1.20 (0.38, 3.65)			
TT + TC/yes	144	250	3.21 (1.77, 5.99)*			
*rs5498/mold*				− 0.16 (− 0.74, 0.43)	0.77 (0.31, 1.89)	− 0.34 (− 1.50, 0.83)
AA/no	220	592	Ref			
GG + GA/no	237	585	1.18 (0.92, 1.51)			
AA/yes	78	135	2.27 (1.52, 3.42)*			
GG + GA/yes	73	135	2.11 (1.41, 3.17)*			
*rs1800925/mold*				0.35 (− 0.03, 0.74)	2.2 (0.75, 6.43)	1.01 (− 0.51, 2.53)
CC/no	318	834	Ref			
TT + CT/no	139	343	1.05 (0.79, 1.38)			
CC/yes	102	192	1.79 (1.28, 2.52)*			
TT + CT/yes	49	78	2.85 (1.72, 4.80)*			
*rs2243250/mold*				− 0.03 (− 0.61, 0.55)	0.94 (0.25, 3.49)	− 0.05 (− 1.04, 0.94)
TT/no	321	792	Ref			
CC + CT/no	136	385	0.80 (0.61, 1.04)			
TT/yes	95	167	1.98 (1.38, 2.85)*			
CC + CT/yes	56	103	1.73 (1.12, 2.69)*			
*rs1801275/mold*				− 0.20 (− 0.88, 0.47)	0.67 (0.19, 2.34)	− 0.35 (− 1.40, 0.70)
AA/no	334	849	Ref			
GG + GA/no	123	328	0.93 (0.70, 1.23)			
AA/yes	106	181	2.13 (1.51, 3.03)*			
GG + GA/yes	45	89	1.72 (1.08, 2.73)*			
*rs324015/mold*				0.39 (− 0.04, 0.81)	5.79 (0.03, 1237.72)	0.73 (− 0.16, 1.61)
CC/no	142	336	Ref			
TT + CT/no	315	841	0.80 (0.61, 1.05)			
CC/yes	35	69	1.35 (0.78, 2.35)			
TT + CT/yes	116	201	1.88 (1.29, 2.74)*			

*SNP* single nucleotide polymorphisms, *Ref* reference, *aOR* adjusted odds ratio, *AP* the attributable proportion due to interaction, *S* the synergy index, *RERI* the relative excess risk due to interaction

Models were adjusted for age, gender, family history of allergy, parental education level, and ETS before and after birth

*FDR adjusted *p* value < 0.05; FDR, False discovery rate

Regarding the IL13 rs1800925 polymorphism, children who carried CC genotype and were exposed to visible mold had a significantly increased risk of childhood asthma with an adjusted OR of 1.79 (95% CI 1.28–2.52), compared to children who carried CC genotype without the exposure. The risk of childhood asthma became higher in children with T allele (aOR 2.85, 95% CI 1.72–4.80) (Table 3). However, RERI, AP, and S were not significant.

For other SNPs, there was no additive interaction between the SNPs and visible mold exposure on childhood asthma under the dominant and recessive models. The combined effects of the SNPs and visible mold exposure were mainly influenced by the visible mold (Table 3 and Additional file 1: Table S6).

## Discussion

Asthma is a complicated disease caused by genes, environmental factors, and their interactions. Our findings indicated that both visible mold exposure and rs7216389 polymorphism increased the risk of childhood asthma. Moreover, the effect of visible mold exposure on asthma became more prominent in children carrying the rs7216389 T allele.

Our finding on mold exposure is consistent with several previous studies [16, 28–32]. For example, the Swedish BAMSE (Barn/Child, Allergy, Milieu, Stockholm, Epidemiology) birth cohort study reported that mold exposure during infancy was associated with asthma and rhinitis up to 16 years old [30]. Cai et al. also found that exposed to visible mold spots was significantly associated with the increased risk of lifetime-ever asthma in 3–6-years-old children in China [16]. Caillaud et al. reviewed papers published from 2006 to 2017 on the effect of indoor mold exposure on asthma and rhinitis. They concluded that there were sufficient evidences on the association between qualitative mold exposure in indoor environments and asthma development, especially in children [31]. Meanwhile, some quantitative assessment of mold exposure studies also found the similar associations [33–36]. Environmental Relative Moldiness Index (ERMI) quantified by 36 indicator-molds in dust samples using DNA-based assays was often used to evaluate the mold level. In a prospective study, Reponen et al. reported children living in a high ERMI value (≥ 5.2) home at 1 year of age had more than twice the risk of developing asthma at the age of 7 years than those in low ERMI value (< 5.2) homes [33]. Furthermore, the authors found that the risk of asthma at age of 7 years was 1.8 times greater for every 10-unit increase in ERMI values in the infants’ homes in a larger cohort study [34].

We also found that the rs7216389 polymorphism was strongly associated with the development of childhood asthma. Rs7216389, a SNP in the GSDMB gene on chromosome 17q12-21, was first linked to childhood asthma in a genome-wide associations study in 2007 [27]. Subsequently, more studies confirmed this association in diverse populations [37–40]. Our results further indicated that the effect was dose-dependent with each C to T substitution at the SNP site. For other SNPs, we did not successfully replicate the risk associations with childhood asthma in our study population. The inconsistency might result from diverse genetic backgrounds in different ethnic populations, different asthma phenotypes, and/or small sample size.

In addition, our study is the first to show an additive interaction between the rs7216389 SNP and visible mold exposure on childhood asthma. Specifically, the mold effect became dramatically greater in subjects carrying the rs7216389 T allele. While this particular finding is new, the gene-environment interaction between rs7216389 genetic variant and exposure to purred pets has been reported. Early cat exposure increased the risk of childhood asthma only in genetically susceptible subjects carrying the rs7216389 high-risk TT genotype [41]. The authors reported that the interaction might be mediated by the expression of orosomucoid-like 3 (ORMDL3), which was modulated by the rs7126389 polymorphism and cat exposure. Similar to cat exposure, mold could activate the expression of ORMDL3 as well.

ORMDL3, adjacent to GSDMB gene, encodes transmembrane proteins localized in the endoplasmic reticulum. It is expressed in multiple cell types including lung structural cells and immune cells [42]. Studies have demonstrated that rs7126389 risk allele (T allele) and Alternaria, which is a common mold in homes, could increase the expression of ORMDL3 in human airway epithelial cells [43–45]. Overexpression of ORMDL3 in airway epithelial activates several downstream pathways including sphingolipids, activating transcription factor 6 (ATF6), sarcoplasmic/endoplasmic reticulum calcium-ATPase (SERCA2b), T-helper 2 cytokines and chemokines [46]. These pathways are closely involved in airway remodeling, hyperresponsiveness and inflammation, which are key features of asthma pathophysiology [42, 47].

Meanwhile, rs7216389 T allele can increase the expression of ORMDL3 in primary immune cells such as CD4+ T cells, where ORMDL3 negatively regulates Interleukin-2 (IL-2) production [48]. Furthermore, IL-2 can modulate the differentiation of CD4+ T helper (Th) cell subsets, including T-helper 1 (Th1), T-helper 2 (Th2), T-helper 17 (Th17), and regulatory T (Treg) cells, the key pathways in allergic and non-allergic inflammation and childhood asthma [49, 50]. Similar to rs7216389 SNP, some fungal components can serve as both allergens and non-allergens and trigger Immunoglobulin E (IgE) response and Th17 response, contributing to the development of asthma and asthma severity [51, 52]. Thus, there could be a synergistic effect between rs7216389 SNP and mold on the development of childhood asthma.

Interleukin (IL)-4/IL-13 pathway genes, including IL-4, IL-13, IL-4 receptor alpha (IL-4Ra), and signal transducer and activator of transcription 6 (STAT6), were frequently linked to serum IgE levels and asthma as they can regulate IL-4 and IL-13 cytokines. IL-4 and IL-13 are the critical cytokines that regulate the switching from Immunoglobulin M (IgM) to IgE in activated B lymphocytes and the differentiation of Th2 cells, which is the pathogenesis of allergic inflammation or childhood asthma [53]. In our study, there was no interaction between these genetic polymorphisms and visible mold exposure on childhood asthma, although they both can trigger the differentiation of Th2 cells. It is possible that the risk of one SNP involved in the IL-4/IL-13 pathway was not sufficient to result in profound changes in asthma susceptibility, just as our genetic associations have shown (see Additional file 1: Table S5) [53]. Larger studies are warranted to investigate the combined effects of the SNPs in the IL-4/IL-13 signaling pathway and the interaction with mold exposure on childhood asthma. Beta-2 adrenergic receptor (ADRB2) gene is located on chromosome 5q31-q32 and encodes β2-adrenergic receptor (β2-AR), which modulates the severity of asthma and the response to β2-AR agonists [54]. Rs1042713 and rs1042714 are two common variants associated with the increased airway responsiveness and the reduced lung function by regulating β2-AR. Therefore, they were more prone to be linked to asthma severity but not asthma [55, 56]. Wang et al. reported that ADRB2 rs1042713 polymorphism significantly interacted with mold odor on asthmatic children with a symptom of night-time awakening, a phenotype of severe asthma. ADRB2 genetic polymorphisms might increase bronchoconstriction which was further accentuated by mold exposure, leading to asthma exacerbation [57]. Severe asthma is a type of asthma that does not respond well to standard asthma treatment. The definition of severe asthma usually relies on the symptoms. Unfortunately, we didn’t have sufficient information to identify the severity of asthmatic children. Therefore, we compared asthmatic and non-asthmatic children without stratifying by severity, which might have led to inconsistent findings.

Our study indicated that indoor mold exposure was associated with an increased risk of childhood asthma. Urbanization has been shown to be associated with asthma and its severity. A probable mechanism was that urbanization could cause the reduction of urban green space, which could protect asthma and other respiratory diseases through reducing environmental pollutants and improving outdoor physical activity [58]. Besides, more and more children might spend their time at home with reduced green space. The indoor mold exposure was more harmful with long-term exposure. As China is one of the biggest developing countries undergoing rapid urbanization, our study indicates that improving indoor air quality may be an important step in preventing childhood asthma in the process of urbanization.

## Strengths and limitations

The current study has several strengths. First, to our knowledge, this is the first study to show an interaction between visible mold exposure and rs7216389 SNP on childhood asthma. Second, asthmatic children were diagnosed by pediatrician according to GINA, reducing the misclassification of outcome.

Moreover, some limitations are worth mentioning. First, mold exposure was ascertained by parents reporting as whether there was visible mold at home, which was subjective and might lead to exposure misclassification. The parents of the case group might have overreported visible mold at home if they knew the potential link between mold and asthma [59]. However, Cai et al. [16] argued that the knowledge of effects of mold on childhood asthma was still limited in Chinese parents. Our own data on self-reported carpet use and pet at home (Table 1) also suggested that reporting bias was likely to be small. On the other hand, mold may be undetectable to the naked eye in spite of being present, resulting in underreporting. In general, subjects tend to underreport household mold growth compared to observations done by trained inspectors or dust mold measurement [60]. Nevertheless, the underreported exposure would more likely to be non-differential in cases and controls. Such a non-differential misclassification of exposure might only lead to an underestimated risk estimate in our study [61]. Mold exposure assessment is difficult and complex. In recent years, some studies used Environmental Relative Moldiness Index (ERMI) to quantify mold contamination in the household. ERMI is a quantitative indicator of 36 indicator-molds in dust samples and is more objective and accurate. Unfortunately, our study did not collect the household dust samples. Second, as in all case–control studies, causality between household visible mold and childhood asthma could not be made. Finally, our sample size was not large enough to study the interaction between SNPs with low allele frequencies and environmental factors. More large studies are needed to explore other gene-mold exposure interaction on childhood asthma.

## Conclusions

In conclusion, we found that visible mold exposure increased the risk of childhood asthma, and a dose-dependent effect of rs7216389 T allele variants on childhood asthma. Moreover, we observed that the effect of visible mold exposure on childhood asthma became more prominent in children carrying the rs7216389 T allele, a gene-environment interaction. Our finding suggests that genetic susceptibility plays a key role in the associations between environmental factors and childhood asthma.

## Supplementary Information

**Additional file 1**. Supplemental Tables and Figure of household mold exposure interacts with inflammation-related genetic variants on childhood asthma.

**Additional file 2**. The role of the SNPs in the inflammation process of asthma.

## Data Availability

The dataset analyzed in the current study is available in the figshare repository, [https://doi.org/10.6084/m9.figshare.14159216.v1].

## Abbreviations

SNP: Single nucleotide polymorphism
GBD: Global Burden of Disease
GWAS: Genome-wide associations
PAMPs: Pathogen-associated molecular patterns
GINA: Global Initiative for Asthma guidelines
ETS: Environmental tobacco smoke
MALDI-TOF: Matrix-assisted laser desorption/ionization time-of-flight
HWE: Hardy–Weinberg equilibrium
OR: Odds ratio
CI: Confidence interval
RERI: Relative excess risks due to interaction
SI: Synergy index
AP: Attributable proportion of interaction
RR: Risk ratio
FDR: False discovery rate
BAMSE: Barn/Child, Allergy, Milieu, Stockholm, Epidemiology
ERMI: Environmental Relative Moldiness Index
ORMDL3: Orosomucoid-like 3
GSDMB: Gasdermin B
ATF6: Activating transcription factor 6
SERCA2b: Sarcoplasmic/endoplasmic reticulum calcium-ATPase
Th1: T-helper 1
Th2: T-helper 2
Th17: T-helper 17
Treg: Regulatory T
IgE: Immunoglobulin E
IL-4: Interleukin-4
IL-13: Interleukin-13
IL-4Ra: Interleukin-4 receptor alpha
STAT6: Signal transducer and activator of transcription 6
IgM: Immunoglobulin M
ADRB2: Beta-2 adrenergic receptor
β2-AR: β2-Adrenergic receptor
