# Supplementary material for: Household mold exposure interacts with inflammation-related genetic variants on childhood asthma: a case–control study
Source: BMC Pulm Med. 2021 Apr 2;21:114. doi: 10.1186/s12890-021-01484-9 (PMC8019181; doi:10.1186/s12890-021-01484-9)
Supplement: Supplementary file 1 — Additional file 1. Supplemental Tables and Figure of household mold exposure interacts with inflammation-related genetic variants on childhood asthma. [file 12890_2021_1484_MOESM1_ESM.docx]

**Supplemental Materials** *for*

**Household mold exposure interacts with inflammation-related**

**genetic variants on childhood asthma: a case-control study**

Yu Zhang^1,2^†, Li Hua^4^†, Quan-Hua Liu^4^, Shu-Yuan Chu^3^, Yue-Xin Gan^2^, Min Wu^5^, Yi-Xiao Bao^4^, Qian Chen^2*^, Jun Zhang^1,2*^

**Supplemental Tables and Figure**

**Table S1.** The information of selected single nucleotide polymorphisms. **Table S2.** Results of primer design. **Table S3.** Four by two table of gene and environmental factor in crossover analysis. **Table S4.** Selected inflammation-related SNPs and minor allele frequencies in study population. **Table S5.** Associations between inflammation-related SNPs and childhood asthma under different genetic models. **Table S6.** Combined effects of SNPs and visible mold exposure on childhood asthma under the recessive genetic model. **Fig S1.** Directed acyclic graph for the interaction between visible mold exposure and SNPs on childhood asthma.

| **Table S1. The information of selected single nucleotide polymorphisms.** | | | |
| --- | --- | --- | --- |
| **SNP** | **Gene** | **Chromosome position** | **Polymorphism** |
| rs1042713 | ADRB2 | 5q31-q32 | A/G |
| rs1042714 | ADRB2 | 5q31-q32 | C/G |
| rs7216389 | GSDMB | 17q21.1 | T/C |
| rs5498 | ICAM1 | 19p13.2 | A/G |
| rs1800925 | IL13 | 5q31.1 | C/T |
| rs2243250 | IL4 | 5q31.1 | T/C |
| rs1801275 | IL4R | 16p11 | A/G |
| rs324015 | STAT6 | 12q13.2-q14 | C/T |
| Definition of abbreviations: SNP = single nucleotide polymorphisms; ADRB2 = beta-2 adrenergic receptor; GSDMB = gasdermin B; ICAM1 = intercellular adhesion molecule-1; IL13 = interleukin 13; IL4 = interleukin 4; IL4R = interleukin-4 receptor; STAT6 = signal transducer and activator of transcription 6. | | | |

| **Table S2. Results of primer design.** | | | |
| --- | --- | --- | --- |
| **SNP_ID** | **1st-PCRP** | **2nd-PCRP** | **UEP_SEQ** |
| rs1042713 | ACGTTGGATGATGACGATGCCCATGCCCA | ACGTTGGATGCAGCGCCTTCTTGCTGGCA | TTGCTGGCACCCAAT |
| rs1042714 | ACGTTGGATGCAGCGCCTTCTTGCTGGCA | ACGTTGGATGATGACGATGCCCATGCCCA | ACACCTCGTCCCTTT |
| rs7216389 | ACGTTGGATGAGTATGAAGTGAGGCAACCC | ACGTTGGATGACCAAGGCCCTTATTAGTGC | GGGCCGAGTCCATGC |
| rs5498 | ACGTTGGATGACTCACAGAGCACATTCACG | ACGTTGGATGAGATCTTGAGGGCACCTAC | AGGGGAGGTCACCCGC |
| rs1800925 | ACGTTGGATGCAACACCCAACAGGCAAATG | ACGTTGGATGAGCCATGTCGCCTTTTCCTG | TTTCCTGCTCTTCCCTC |
| rs2243250 | ACGTTGGATGTAACAGGCAGACTCTCCTAC | ACGTTGGATGTGATACGACCTGTCCTTCTC | AACTTGGGAGAACATTGT |
| rs1801275 | ACGTTGGATGACCCTGCTCCACCGCATGTA | ACGTTGGATGATCCTCCGCCGAAATGTCCT | GCCCCACCAGTGGCTATC |
| rs324015 | ACGTTGGATGCTCAGAGAGCTCTGTATGTG | ACGTTGGATGTGGAAGGGAAGTTCAGGCTC | GGGGTCAGGCTCTGAGACAC |
| 1st-PCRP: Polymerase chain reaction (PCR) amplification primer, forward primer sequence.  2nd-PCRP: Polymerase chain reaction (PCR) amplification primer, reverse primer sequence.  UEP_SEQ: Unextended primer sequence.  The 10nt sequence ACGTTGGATG was added to the 5′ end of each PCR primer so that they will not interfere in mass spectra. | | | |

| **Table** S3. Four by two table of gene and environmental factor in crossover analysis. | | | | | |
| --- | --- | --- | --- | --- | --- |
| **G** | **E** | **Cases** | **Controls** | **OR** | **M**eaning |
| 0 | 0 | a | b | OR00 = 1 | Reference |
| 1 | 0 | c | d | OR10 = ad/bc | Independent effect of G |
| 0 | 1 | e | f | OR01 = af/be | Independent effect of E |
| 1 | 1 | g | h | OR11 = ah/bg | Joint effect of G and E |
| Definition of abbreviations: G = risk genotype; E = environmental factor; OR = odds ratio; 0 and 1 represent absence and presence of the risk factors, respectively. | | | | | |

| **Table S4. Selected inflammation-related SNPs and minor allele frequencies in study population.** | | | | |
| --- | --- | --- | --- | --- |
| **Gene** | **SNP** | **Alleles^a^** | **Minor allele frequency** | **P value for HWE**^b^ |
| ADRB2 | rs1042713 | A/G | 0.44 | 0.18 |
| ADRB2 | rs1042714 | C/G | 0.10 | 0.12 |
| GSDMB | rs7216389 | T/C | 0.24 | 0.54 |
| ICAM1 | rs5498 | A/G | 0.29 | 0.35 |
| IL13 | rs1800925 | C/T | 0.16 | 1.00 |
| IL4 | rs2243250 | T/C | 0.18 | 0.51 |
| IL4R | rs1801275 | A/G | 0.16 | 0.90 |
| STAT6 | rs324015 | C/T | 0.48 | 0.30 |
| Definition of abbreviations: SNP = single nucleotide polymorphisms; HWE = Hardy Weinberg equilibrium; ADRB2 = beta-2 adrenergic receptor; GSDMB = gasdermin B; ICAM1 = intercellular adhesion molecule-1; IL13 = interleukin 13; IL4 = interleukin 4; IL4R = interleukin-4 receptor; STAT6 = signal transducer and activator of transcription6.  ^a^ Major allele/Minor allele.  ^b^ Hardy Weinberg equilibrium was estimated for the control group. | | | | |

| **Table S5. Associations between inflammation-related SNPs and childhood asthma under different genetic models.** | | | | | | | | |  |
| --- | --- | --- | --- | --- | --- | --- | --- | --- | --- |
| **SNP** | **Recessive model** | |  | **Dominant model** | |  | **Additive model** | | |
|  | **Genotype** | **aOR (95% CI)** |  | **Genotype** | **aOR (95% CI)** |  | **Model**^b^ | **aOR (95% CI)** | |
| rs1042713 | AG+AA | ref |  | AA | ref |  | G allele | 1.05 (0.91, 1.22) | |
|  | GG | 1.00 (0.76, 1.3) |  | GG+GA | 1.13 (0.90, 1.41) |  |  |  |  |
| rs1042714 | CG+CC | ref |  | CC | ref |  | G allele | 0.97 (0.75, 1.25) | |
|  | GG | 1.98 (0.56, 7.84) |  | GG+GC | 0.94 (0.71, 1.23) |  |  |  |  |
| rs7216389^a^ | CC+CT | ref |  | CC | ref |  | T allele^c^ | 1.32 (1.11, 1.57)* | |
|  | TT | 1.34 (1.08, 1.66) |  | TT+TC | 1.72 (1.09, 2.77) |  |  |  |  |
| rs5498 | AG+AA | ref |  | AA | ref |  | G allele | 1.10 (0.93, 1.3) | |
|  | GG | 1.19 (0.80, 1.77) |  | GG+GA | 1.10 (0.89, 1.36) |  |  |  |  |
| rs1800925 | CT+CC | ref |  | CC | ref |  | T allele | 1.13 (0.92, 1.39) | |
|  | TT | 1.09 (0.54, 2.16) |  | TT+TC | 1.15 (0.91, 1.45) |  |  |  |  |
| rs2243250 | CT+TT | ref |  | TT | ref |  | C allele | 0.88 (0.73, 1.07) | |
|  | CC | 0.96 (0.52, 1.74) |  | CC+CT | 0.85 (0.68, 1.07) |  |  |  |  |
| rs1801275 | AG+AA | ref |  | AA | ref |  | G allele | 0.94 (0.77, 1.15) | |
|  | GG | 1.12 (0.59, 2.08) |  | GG+GA | 0.91 (0.72, 1.15) |  |  |  |  |
| rs324015 | CT+CC | ref |  | CC | ref |  | T allele | 0.90 (0.78, 1.05) | |
|  | TT | 0.83 (0.64, 1.06) |  | TT+TC | 0.91 (0.72, 1.15) |  |  |  |  |
| Definition of abbreviations: aOR = adjusted odds ratio; ref = reference genotype coded as 0; CI = confidence interval; SNP = single nucleotide polymorphism.  Models were adjusted for gender and age.  ^a^ The reference allele of each SNPs was major allele, except for rs7216389 SNP; C allele, the minor allele of rs7216389 SNP, was defined as reference allele because of the low risk effect.  ^b^ Additive genetic model, the genotypes were categorized into a three-level variable for the number of minor alleles (0,1,2).  ^c^ The genotypes of rs7216389 SNP were categorized into a three-level variable for the number of major alleles under the additive model (0,1,2).  * FDR adjusted P value<0.05, FDR, False discovery rate. | | | | | | | | | |

| **Table S6. Combined effects of SNPs and visible mold exposure on childhood asthma under the recessive genetic model.** | | | | | | |
| --- | --- | --- | --- | --- | --- | --- |
| **SNPs/ mold exposure** | **No. of asthma cases** | **Group size** | **OR (95% CI)** | **AP** | **S** | **RERI** |
| **rs1042713/ mold** |  |  |  | 0.24 (-0.28, 0.76) | 1.69 (0.48, 5.90) | 0.59 (-0.99, 2.17) |
| AA+AG/no | 367 | 941 | ref |  |  |  |
| GG/no | 90 | 236 | 0.95 (0.69, 1.29) |  |  |  |
| AA+AG/yes | 121 | 220 | 1.91 (1.39, 2.62)* |  |  |  |
| GG/yes | 30 | 50 | 2.44 (1.33, 4.57)* |  |  |  |
| **rs7216389/ mold** |  |  |  | -0.12 (-0.68, 0.44) | 0.83 (0.37, 1.86) | -0.30 (-1.59, 1.00) |
| CT+CC/no | 170 | 501 | ref |  |  |  |
| TT/no | 287 | 676 | 1.34 (1.04, 1.73) |  |  |  |
| CC+CT/yes | 59 | 106 | 2.37 (1.51, 3.73)* |  |  |  |
| TT/yes | 92 | 164 | 2.41 (1.65, 3.55)* |  |  |  |
| **rs5498/ mold** |  |  |  | -0.22 (-1.27, 0.83) | 0.68 (0.11, 4.11) | -0.41 (-2.09, 1.28) |
| AA+GA/no | 422 | 1095 | ref |  |  |  |
| GG/no | 35 | 82 | 1.21 (0.74, 1.97) |  |  |  |
| AA+GA/yes | 135 | 241 | 2.07 (1.53, 2.80)* |  |  |  |
| GG/yes | 16 | 29 | 1.87 (0.85, 4.21) |  |  |  |
| **rs2243250/ mold** |  |  |  | -0.26 (-2.32, 1.80) | 0.60 (0.01, 37.27) | -0.43 (-3.15, 2.30) |
| TT+TC/no | 442 | 1137 | ref |  |  |  |
| CC/no | 15 | 40 | 1.03 (0.50, 2.04) |  |  |  |
| TT+TC/yes | 147 | 263 | 2.03 (1.52, 2.72)* |  |  |  |
| CC/yes | 4 | 7 | 1.63 (0.33, 8.85) |  |  |  |
| **rs1801275/ mold** |  |  |  | 0.37 (-0.63, 1.37) | 2.2 (0.19, 24.91) | 1.16 (-3.70, 6.01) |
| AA+AG/no | 443 | 1143 | ref |  |  |  |
| GG/no | 14 | 34 | 0.98 (0.45, 2.04) |  |  |  |
| *Continued to the next page* | | | | | | |
| *Table S6. Continued* | | | | | | |
| AA+AG/yes | 146 | 262 | 1.99 (1.49, 2.67)* |  |  |  |
| GG/yes | 5 | 8 | 3.13 (0.69, 16.58) |  |  |  |
| **rs324015/ mold** |  |  |  | -0.32 (-1.10, 0.46) | 0.65 (0.27, 1.57) | -0.77 (-2.60, 1.05) |
| CC+TC/no | 97 | 280 | ref |  |  |  |
| TT/no | 360 | 897 | 1.32 (0.98, 1.79) |  |  |  |
| CC+TC/yes | 33 | 57 | 2.87 (1.55, 5.38)* |  |  |  |
| TT/yes | 118 | 213 | 2.42 (1.64, 3.58)* |  |  |  |
| Definition of abbreviations: SNP = single nucleotide polymorphisms; ref=reference; OR = odds ratio; AP = the attributable proportion due to interaction; S = the synergy index; RERI = the relative excess risk due to interaction.  Models were adjusted for age, gender, family history of allergy, parental education level, and ETS before and after birth.  Rs1042714 and rs1800925 SNPs were not performed because of small number of children carrying minor allele homozygotes.  *FDR adjusted P-value<0.05; FDR, False discovery rate. | | | | | | |


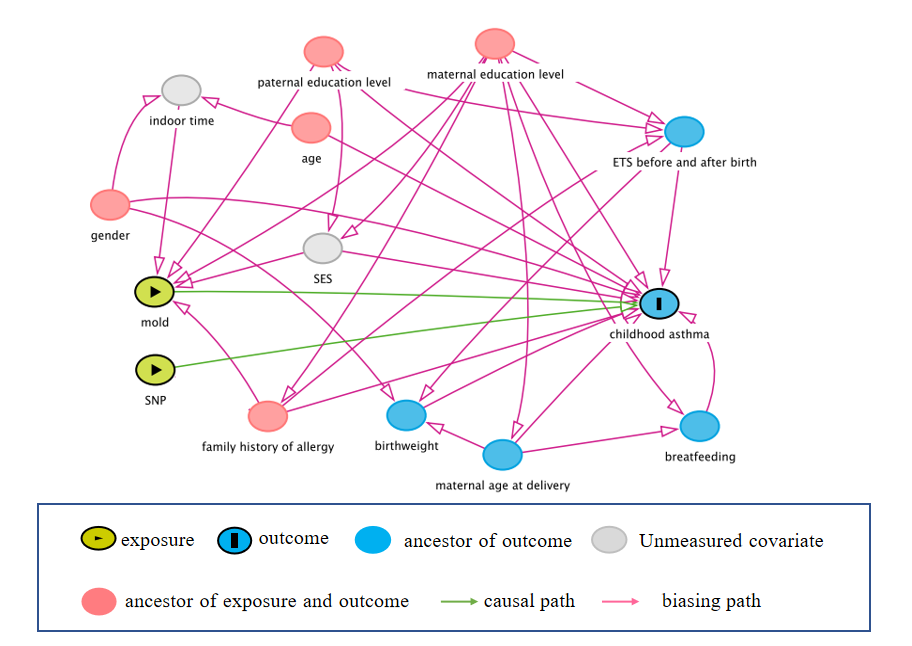


Fig. S1. Directed acyclic graph for the interaction between visible mold exposure and SNPs on childhood asthma. SNP: single nucleotide polymorphisms, SES: [socioeconomic status](C:/Users/zhangyu/Desktop/javascript:;). DAGitty version 3.0.
