# Supplementary material for: Household mold exposure interacts with inflammation-related genetic variants on childhood asthma: a case–control study
Source: BMC Pulm Med. 2021 Apr 2;21:114. doi: 10.1186/s12890-021-01484-9 (PMC8019181; doi:10.1186/s12890-021-01484-9)
Supplement: Supplementary file 2 — Additional file 2. The role of the SNPs in the inflammation process of asthma. [file 12890_2021_1484_MOESM2_ESM.docx]

**Supplemental Materials** *for*

**Household mold exposure interacts with inflammation-related**

**genetic variants on childhood asthma: a case-control study**

Yu Zhang^1,2^†, Li Hua^4^†, Quan-Hua Liu^4^, Shu-Yuan Chu^3^, Yue-Xin Gan^2^, Min Wu^5^, Yi-Xiao Bao^4^, Qian Chen^2*^, Jun Zhang^1,2*^

**1. The role of the SNPs in the inflammation process of asthma**

**1.1 ADRB2 genetic variants:**

Beta-2 adrenergic receptor (ADRB2) gene, an intronless gene, is located on chromosome 5q31-q32 and encodes the β2-adrenergic receptor (β2-AR). β2-AR abundantly expresses on smooth muscle cells and is associated with airway hypersensitivity (AHR), asthma severity, and the response to medications[1]. Recently, group 2 innate lymphoid cells (ILC2s) were linked to allergic asthma by the production of classical type 2 cytokines in human lung tissue, such as IL4, IL5, and IL13 [2]. Moriyama et al. found that β2-AR negatively regulated ILC2 responses and type 2 inflammation after exposure to allergens [3]. Rs1042713 and rs1042714 polymorphisms, located in the coding region of the ADRB2 gene, can change the amino acid sequence, further leading to down-regulation of the β2-AR [4]. β2-AR down-regulation may further cause AHR, lung function, and allergic inflammation.

**1.2 ICAM1 genetic variant:**

Intercellular adhesion molecule 1 (ICAM1) gene is located on chromosome 19p13.3-p13.2 and encodes ICAM1 protein. ICAM1 plays a central role in the recruitment and migration of leukocytes to sites of inflammation and is linked to childhood asthma [5]. ICAM1 rs5498 polymorphism can influence protein-protein dimerization and consequently alter the ability to bind inflammatory cells. Moreover, the rs5498 polymorphism may make ICAM1 more prone to enzymatic cleavage and further decrease the binding ability of leukocytes [6]. In a word, rs5498 polymorphism can alter the binding capacity of leukocytes and influence the development of airway inflammation.

**1.3 GSDMB genetic variant:**

Rs7216389, an SNP located in the first intron of the gasdermin B (GSDMB) gene, is significantly associated with increased orosomucoid-like 3 (ORMDL3) expression in human airway epithelial and primary immune cells. Overexpression of ORMDL3 in airway epithelial can activate several downstream pathways including sphingolipids, activating transcription factor 6 (ATF6), sarcoplasmic/endoplasmic reticulum calcium-ATPase (SERCA2b), T-helper 2 cytokines, and chemokines. These pathways are closely involved in airway remodeling, hyperresponsiveness, and inflammation [7]. Furthermore, ORMDL3 in primary immune cells can negatively regulate Interleukin-2 (IL-2) production, which influences the differentiation of CD4+ T helper (Th) cell subsets, resulting in allergic or non-allergic inflammation [8].

**1.4 IL-4/IL-13 pathway genetic variants:**

Interleukin (IL)-4/IL-13 pathway genes are mainly composed of IL-4, IL-13, IL-4 receptor alpha (IL-4Ra), and signal transducer and activator of transcription 6 (STAT6), which encode the corresponding cytokines. These genes regulate the differentiation of naïve CD4+ into a Th2-cell polarized effector phenotype, a key biological process in allergic inflammation and asthma [9]. IL-4 or IL-13 activates IL-4Ra and bounds to IL-4/IL-13/IL-4Ra, leading to the phosphorylation of STAT6 through the Janus tyrosine kinase. Phosphorylated STAT6 then activates the transcription of target genes and induces IgE switching and proinflammatory activation [10]. For IL-4 rs2243250 polymorphism, it has been recognized on upstream of the transcription initiation site. There was evidence that the polymorphic T allele enhances the binding of a transcription factor that results in overexpression of the IL-4 gene, further increasing the strength of any IL-4 dependent allergic inflammation [11]. Rs1800925 is located in the promoter region of the IL-13 gene and affects the expression of IL-13 through the similar pathway of IL-4 rs2243250. Rs302415 polymorphism in the 3’-untranslated region of the STAT6 gene is linked to asthma in Chinese [12]. It has been shown that rs302415 polymorphism is significant in linkage disequilibrium with 13 GT repeat polymorphism of STAT6 exon 1. It influences STAT6 gene expression by affecting the translation, coding capacity, mRNA stability, and localization of the mRNA in the cytoplasm [13]. Rs1801275 polymorphism, located in the exonic region of the IL-4R gene, has been reported to influence the expression of IL-4/IL-13 pathway genes response by affecting the binding of STAT6, and further induces allergic inflammation [14]. Meanwhile, rs1801275 polymorphism increases the recruitment of a growth-factor-receptor-bound protein 2 (GRB2), leading to airway inflammation by driving IL-4-directed iTreg cell differentiation towards the Th17 cell lineage [15].

**References**

1. Liang SQ, Chen XL, Deng JM, Wei X, Gong C, Chen ZR, Wang ZB: **Beta-2 adrenergic receptor (ADRB2) gene polymorphisms and the risk of asthma: a meta-analysis of case-control studies**. *PLoS One* 2014, **9**(8):e104488.

2. van der Ploeg EK, Carreras Mascaro A, Huylebroeck D, Hendriks RW, Stadhouders R: **Group 2 Innate Lymphoid Cells in Human Respiratory Disorders**. *J Innate Immun* 2020, **12**(1):47-62.

3. Moriyama S, Brestoff JR, Flamar AL, Moeller JB, Klose CSN, Rankin LC, Yudanin NA, Monticelli LA, Putzel GG, Rodewald HR *et al*: **β(2)-adrenergic receptor-mediated negative regulation of group 2 innate lymphoid cell responses**. *Science* 2018, **359**(6379):1056-1061.

4. Guo X, Zheng H, Mao C, Guan E, Si H: **An association and meta-analysis study of 4 SNPs from beta-2 adrenergic receptor (ADRB2) gene with risk of asthma in children**. *Asian Pac J Allergy Immunol* 2016, **34**(1):11-20.

5. Li YF, Tsao YH, Gauderman WJ, Conti DV, Avol E, Dubeau L, Gilliland FD: **Intercellular adhesion molecule-1 and childhood asthma**. *Hum Genet* 2005, **117**(5):476-484.

6. Klaassen EM, van de Kant KD, Jobsis Q, Penders J, van Schooten FJ, Quaak M, den Hartog GJ, Koppelman GH, van Schayck CP, van Eys G *et al*: **Integrative genomic analysis identifies a role for intercellular adhesion molecule 1 in childhood asthma**. *Pediatr Allergy Immunol* 2014, **25**(2):166-172.

7. Das S, Miller M, Broide DH: **Chromosome 17q21 Genes ORMDL3 and GSDMB in Asthma and Immune Diseases**. *Adv Immunol* 2017, **135**:1-52.

8. Schmiedel BJ, Seumois G, Samaniego-Castruita D, Cayford J, Schulten V, Chavez L, Ay F, Sette A, Peters B, Vijayanand P: **17q21 asthma-risk variants switch CTCF binding and regulate IL-2 production by T cells**. *Nat Commun* 2016, **7**:13426.

9. Vercelli D: **Discovering susceptibility genes for asthma and allergy**. *Nat Rev Immunol* 2008, **8**(3):169-182.

10. Kabesch M, Schedel M, Carr D, Woitsch B, Fritzsch C, Weiland SK, von Mutius E: **IL-4/IL-13 pathway genetics strongly influence serum IgE levels and childhood asthma**. *J Allergy Clin Immunol* 2006, **117**(2):269-274.

11. Rosenwasser LJ, Klemm DJ, Dresback JK, Inamura H, Mascali JJ, Klinnert M, Borish L: **Promoter polymorphisms in the chromosome 5 gene cluster in asthma and atopy**. *Clinical & Experimental Allergy* 1995, **25**(s2):74-78.

12. Zhu L, Zhu Q, Zhang X, Wang H: **The correlation analysis of two common polymorphisms in STAT6 gene and the risk of asthma: a meta-analysis**. *PLoS One* 2013, **8**(7):e67657.

13. Tamura K, Suzuki M, Arakawa H, Tokuyama K, Morikawa A: **Linkage and association studies of STAT6 gene polymorphisms and allergic diseases**. *Int Arch Allergy Immunol* 2003, **131**(1):33-38.

14. Tachdjian R, Mathias C, Al Khatib S, Bryce PJ, Kim HS, Blaeser F, O'Connor BD, Rzymkiewicz D, Chen A, Holtzman MJ *et al*: **Pathogenicity of a disease-associated human IL-4 receptor allele in experimental asthma**. *J Exp Med* 2009, **206**(10):2191-2204.

15. Massoud AH, Charbonnier LM, Lopez D, Pellegrini M, Phipatanakul W, Chatila TA: **An asthma-associated IL4R variant exacerbates airway inflammation by promoting conversion of regulatory T cells to TH17-like cells**. *Nat Med* 2016, **22**(9):1013-1022.
